# Effect of *Linum usitatissimum* L. (linseed) oil on mild and moderate carpal tunnel syndrome: a randomized, double-blind, placebo-controlled clinical trial

**DOI:** 10.1186/2008-2231-22-43

**Published:** 2014-05-21

**Authors:** Mohammad Hashem Hashempur, Kaynoosh Homayouni, Alireza Ashraf, Alireza Salehi, Mohsen Taghizadeh, Mojtaba Heydari

**Affiliations:** 1 Research Center for Traditional Medicine and History of Medicine, Shiraz University of Medical Sciences, Shiraz, Iran; 2 Essence of Parsiyan Wisdom Institute, Traditional Medicine and Medicinal Plant Incubator, Shiraz University of Medical Sciences, Shiraz, Iran; 3 Department of Physical Medicine and Rehabilitation, Shiraz University of Medical Sciences, Shiraz, Iran; 4 Shiraz Geriatric Research Center, Shiraz University of Medical Sciences, Shiraz, Iran; 5 Shiraz Burn Research Center, Shiraz University of Medical Sciences, Shiraz, Iran; 6 Research Center for Biochemistry and Nutrition in Metabolic Disease, Kashan University of Medical Sciences, Kashan, Iran

**Keywords:** Carpal tunnel syndrome, *Linum usitatissimum*, Linseed oil, Iranian traditional medicine, Randomized controlled trial, Herbal medicine, Complementary therapies

## Abstract

**Background:**

Carpal tunnel syndrome is known as the most common entrapment neuropathy. Conservative treatments cannot reduce the symptomatic severity satisfactorily; therefore, effectiveness of *Linum usitatissimum* L. (linseed) oil on carpal tunnel syndrome, as a complementary treatment, was evaluated in the current study. Linseed oil is a well-known preparation in Iranian traditional medicine and its analgesic, anti-inflammatory and anti-oxidative effects have been shown in previous studies.

**Methods:**

A randomized, double-blind, placebo-controlled clinical trial was conducted. One hundred patients (155 hands) with idiopathic mild to moderate carpal tunnel syndrome aged between 18 and 65 years old were randomized in two parallel groups. These two groups were treated during 4 weeks with topical placebo and linseed oil. In addition, a night wrist splint was prescribed for both groups. Symptomatic severity and functional status were measured using Boston Carpal Tunnel Questionnaire. In addition, median sensory nerve conduction velocity, motor distal latency, sensory distal latency and compound latency as electrodiagnostic parameters were measured at baseline and after the intervention period.

**Results:**

After the intervention, significant improvement was observed regarding Boston Carpal Tunnel Questionnaire symptomatic severity and functional status mean differences (*p* <0.001) in the linseed oil group compared with those in the placebo group. Also, regarding the mean differences of both groups, significant improvement of nerve conduction velocity of the median nerve was seen in the linseed oil group by a value of 2.38 m/sec (*p* < 0.05). However, motor distal latency and sensory distal latency of the median nerve showed no between-group significant changes (*p* = 0.14 for both items). Finally, compound latency was improved slightly in the case group, comparing mean differences between the groups (*p* <0.05). No significant adverse events were reported from using linseed oil.

**Conclusions:**

It seems that linseed oil could be effective in the management of mild and moderate carpal tunnel syndrome, especially in improving the severity of symptoms and functional status. In addition, its effect on electerodiagnostic parameters, especially on the nerve conduction velocity, can be considered as a valuable point.

## Background

Carpal tunnel syndrome (CTS), a condition in which the median nerve compression occurs, is known as the most common entrapment neuropathy [1]. Various treatment options, as surgical and non-surgical, have been suggested for CTS. Surgery is usually considered for patients with an experience of conservative treatment failure and those who have severe CTS [2,3], while non-surgical treatments are usually prescribed as an initial option for the patients who do not have any evidence of denervation in electromyography, cannot undergo surgery, or suffer from non-constant symptoms of mild to moderate CTS [4,5]. Standard non-surgical treatments vary from exercise and activity modification to wrist splinting (as the most frequently reported treatment [6]), use of oral medications like NSAIDs and corticosteroids, and even locally injected steroids [7,8]. However, conservative treatments cannot reduce symptomatic severity satisfactorily [9-11]; therefore, new conservative treatments are needed to be evaluated in randomized controlled trials.

Nowadays, complementary and alternative medicine (CAM) is welcomed by general population, governments and World Health Organization. Easy accessibility, lower costs and origination of them from nature are the main causes of this worldwide popularity [12]. Therefore, CAM treatments can play an important role as new conservative treatments for CTS.

*Linum usitatissimum* L. (from the family Linaceae), commonly known as flax or linseed, is a herb that is native to Europe, Asia and Mediterranean regions [13]. Linseed oil or flaxseed oil is obtained from its dried ripe seeds. In addition to edible uses of this oil, it is known as an anti-inflammatory [14,15] antioxidant [16,17] and analgesic [18] oil. Therefore, due to the mentioned beneficial properties, it is used in several studies on a variety of subjects such as arthritis [15], dermatologic complaints [19], breast cancer [20] and even keratoconjunctivitis [21].

The topical use of linseed oil has been approved for a variety of skin disorders [22]. For instance, the Brazilian national pharmacopoeia has approved its topical administration in cases with pruritus, and in patients of burn [13]. In addition, some studies examined the topical usage of this compound in animal model of skin wound healing, and in the prevention of peri-ileostomy skin excoriation. No toxicity was reported in such studies [23].

Linseed oil could play an anti-inflammatory role when used by different routes of administration in animal models. In fact, its inhibition on prostaglandin E_2_, leukotriene B_4_, histamine and bradykinin can make it a potent anti-inflammatory agent against distinct phases of inflammation, comparable with standard aspirin [24]. In addition, it seems that analgesic activity of linseed oil is peripherally mediated [18]. Analgesic activity of linseed oil may be due to a combination of its inhibitory effect of prostaglandin, histamine, bradykinin and acetylcholine [25].

In addition, linseed oil is a well-known and frequently-used medicine in Iranian traditional medicine (ITM). According to the most famous and reliable ITM books, i.e. Avicenna’s Canon of Medicine and Liber Continens of Rhazes, linseed oil can be used as an analgesic and anti-fibrosis drug [26,27].

Therefore, according to ITM concepts and some unpublished experiences by experts about potential effect of linseed oil on CTS and also supporting data about some properties of linseed oil (e.g. analgesic, antioxidant and anti-inflammatory), we hypothesized its possible effect for CTS. Therefore, the current study aimed to assess the effectiveness of topical flaxseed oil in patients with mild and moderate CTS.

## Materials and methods

### Study design

The study was designed as a two-arm, randomized, placebo-controlled, double- blind clinical trial using a parallel design.

### Ethical considerations

The study protocol was in compliance with the Declaration of Helsinki (1989 revision) and approved by the Local Medical Ethics Committee of Shiraz University of Medical Sciences (SUMS) with reference number: CT-92-6709. The trial was registered in Iranian Registry of Clinical Trials (registration ID: IRCT2012103111341N1). A written informed consent was signed by all of the enrolled participants.

### Preparation of test drug, placebo and wrist splint

Seeds of the test drug were purchased from the local market and authenticated by a botanist at Kashan University of Medical Sciences. A voucher specimen was preserved for future reference.

The seeds were coarsely ground in an environment of mild heat, and then cold-pressed (35°C). The extracted oil was subjected to gas chromatographic (GC) analysis. The findings revealed the presence of major components as linolenic acid (54.2%), oleic acid (20.39%), linoleic acid (12.26%), palmitic acid (5.99%), and stearic acid (5.7%). Nitrogen purging was carried out to avoid oxidation. In addition, the bottles had no head space (the oil was filled to the bottle brim).

Pharmaceutical graded paraffin was considered as the placebo. In addition, standard coloring agent in a little amount and standard range was used to make paraffin’s color similar to that of the linseed oil.

**Figure 1:**
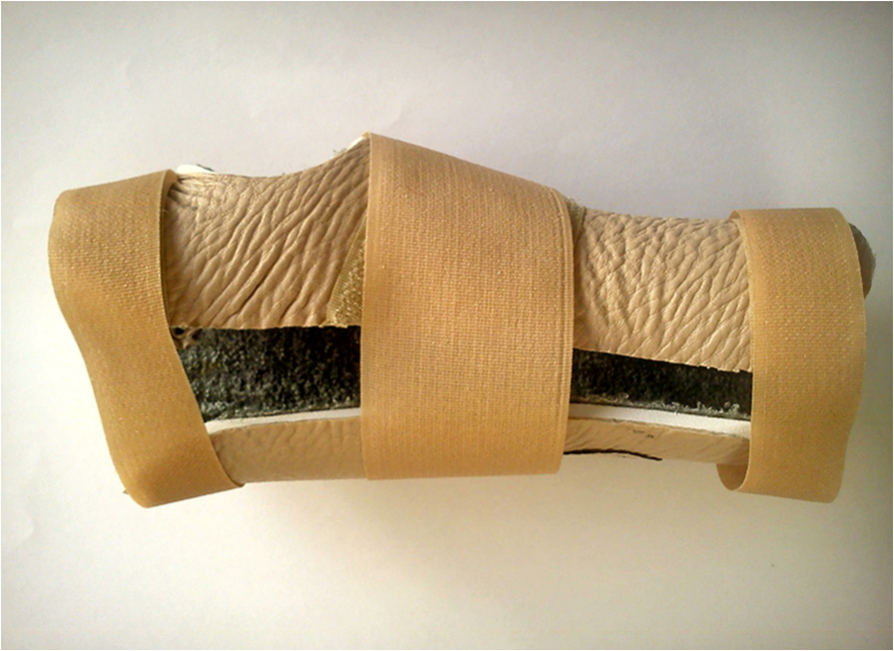
**A photograph of fabricated wrist splint that was prescribed for all of the participants (dorsal view).**

A wrist splint (Dr. K. H.®) immobilized the wrist in an extension position (external angle: 20° and internal angle: 5°). The splint was made of 5-mm medical foam, lined internally with fabric and externally with thin leather. In addition, it had three adjustable Velcro fastenings on its dorsal side (Figure 1).

### Inclusion and exclusion criteria

Patients (from the Outpatient Clinic of Shahid Faghihi Hospital, an academic teaching center, affiliated with SUMS) with suspected CTS (according to history and physical examination) were selected after electrophysiologic confirmation study.

Detailed inclusion and exclusion criteria are shown in Table 1. The eligibility criteria included briefly: patients of both sexes aged between 18 and 65 years old, with idiopathic mild and moderate CTS. Some of the most important exclusion criteria were: coexisting serious illness, rheumatoid arthritis, CTS related to systemic diseases and pregnancy. In addition, the patients were excluded if they had previous surgery for CTS or intracarpal steroid injections.

**Table 1:** Inclusion and exclusion criteria

**Inclusion criteria**	Patients of both sexes aged between 18 and 65 years old willing to sign the informed consent form
	Clinical symptoms and signs of CTS (at least 2 symptoms or 1 sign plus 1 symptom^37^), including:
	- Pain
	- Paraesthesia
	- Hypoesthesia
	- Numbness
	- Tingling
	- Positive Phalen test
	- Positive Tinnel test
	Electrodiagnostic evidence of mild and moderate idiopathic CTS, including:
	● SDL > 3.7 m Sec
	● SNCV < 40 m/Sec
	● MDL > 4.2 m Sec
	● CL > 2.4 m Sec
**Exclusion criteria**	Positive history of hypersensitivity to linseed oil
	Inability of data gathering forms completion (such as cognitive impairment or language problem)
	Patients with severe CTS, clinical and electrodiagnostic evidence including (if any of these evidences was found):
	– Thenar atrophy
	–Fibrillation potentials or reinnervation on needle EMG of APB muscle
	–Electrophisiologic study:
	● SDL > 5.3 m Sec OR Absent
	● SNCV < 28 m/Sec
	● MDL > 6.5 m Sec OR Absent
	● CL > 3.2 m Sec
	Coexisting cervical radiculopathies, brachial plexopathies or more proximal median mononeuropathies
	Clinical and electrophysiological signs of polyneuropathy
	Rheumatologic diseases, like RA, systemic sclerosis, SLE and amyloidosis
	Endocrinologic diseases, such as DM and hypothyroidism
	Conditions that can mimic CTS such as multiple sclerosis
	Pregnancy
	Coexisting serious illness, such as renal and heart failure
	Recent or ongoing inevitable use of corticosteroids or analgesics
	Previous surgery for CTS
	Intra-articular injection within the previous 6 months
	Positive history of severe trauma or fracture of wrist bones

SDL: sensory distal latency; SNCV: sensory nerve conduction velocity; MDL: motor distal latency; CL: compound latency; EMG: electromyography; APB: abductor policis brevis; RA: rheumatoid arthritis; SLE: systemic lupus erythematosus; DM: diabetes mellitus.

### Electrodiagnosis

The electrophysiologic assessments were performed by a “MEDLEC SYNERGY VIASIS” electromyography device with two 6 mm felt tips bar electrodes as the stimulators and recorders (diameter of pads 23 mm apart). Median distal motor latency was measured by a bipolar stimulating electrode at the wrist and a bipolar surface-recording located on the abductor pollicis brevis muscle (8 cm from stimulus electrodes at the wrist). Antidromic sensory nerve action potentials evoked at the wrist were recorded from the middle finger. Standard distances (7 cm from recorder, at mid palm and 14 cm from recorder at wrist) were kept between the stimulator and recorder electrodes. For recording the compound nerve action potential, stimulation at mid palm and recording at wrist (7 cm apart) was performed. In addition, using a concentric needle electrode, electromyography was performed on the abductor pollicis brevis muscle. We defined denervation as sustained, abnormal spontaneous activity. This ranged from 0 to 4+, in the form of positive waves or fibrillations. The skin temperature during all of the electrodiagnostic studies was at least 31°C and all of the assessments were carried out in a similar constant room temperature between 23°C and 25°C. In addition, due to the possibility of diurnal variation in clinical and electrophysiologic assessments [28], all of the patients were assessed at the follow up visit at a similar time of the day as the first session. In addition, the patients were assessed on their visits by the same authors (K.H and A.A) who performed clinical examinations and electrodiagnostic assessments and were blinded about each patient’s allocation.

### Intervention

#### Splint

All of the patients were prescribed to use the wrist splint during the study period. In fact, the test drug and placebo were added to this standard treatment. The wrist splint prescription was night-only because of symptoms usually worsening at night, in addition to obtaining a higher compliance, considering the patient’s concern about splint’s interference with his/her daily activities.

#### Placebo and test drug

Both of the drug and placebo were prescribed to be used in the morning and evening time for a period of 4 weeks, 5 drops per use, topically on the palmar wrist territory. The patients were advised not to massage the mentioned zone.

### Outcome assessment

Boston Carpal Tunnel Questionnaire (BCTQ), as a self-administered, validated measurement, was the primary outcome measure. The BCTQ assesses symptom severity score (BCTQ SYMPT) and functional status score (BCTQ FUNCT). These scores are evaluated by an eleven-item scale and an eight-item scale, respectively [29]. The items of each scale consist of multiple-choice responses from 1 (as the mildest) to 5 (as the most severe). The BCTQ SYMPT and FUNCT are calculated as the mean of the scores for the individual items.

We used the Persian version of BCTQ that was validated previously, showing to have a reasonable reliability, sensitivity and internal consistency [30].

Secondary outcome measures were median nerve sensory distal latency (SDL), sensory nerve conduction velocity (NCV), motor distal latency (MDL) and compound latency (CL).

At the beginning of the enrollment and after 4 weeks of intervention, the data related to both primary and secondary outcome measures were obtained and recorded on the patient’s data form. We excluded the patients who had recent or ongoing inevitable use of corticosteroids or analgesics; however, the included patients were asked to record their use of analgesics as the rescue drug.

### Randomization, blinding and concealment of allocation

The eligible patients were randomly allocated to two parallel groups, the drug and placebo groups, by the secretary of the clinic. She was trained and instructed to use a block-randomization list (non-stratified, with the same block lengths, generated by computer) sequentially. In the case of patients affected by CTS bilaterally, both wrists were allocated to the same intervention (i.e. drug or placebo). The physicians, researchers, and statisticians were blind to the allocation of patients. Moreover, due to the same shape and size of the drug and placebo containers and similarity in color, the patients were blind to their allocation.

### Statistical analysis

The intention-to-treat population used in all of the analyses included all randomized patients who completed their follow up, whether adhering completely to the clinical trial protocol or not.

Demographic and clinical characteristics of the participants were shown as the mean ± standard deviation (SD) for continuous variables. Differences of pre- and post-treatment were reported as mean and 95% confidence intervals.

Normality for continuous variables was checked using Kolmogorov-Smirnov test. Data were analyzed using Chi-square test, independent and paired samples t-test, and Mann–Whitney test. *P* values less than 0.05 were considered statistically significant.

## Results

**Figure 2:**
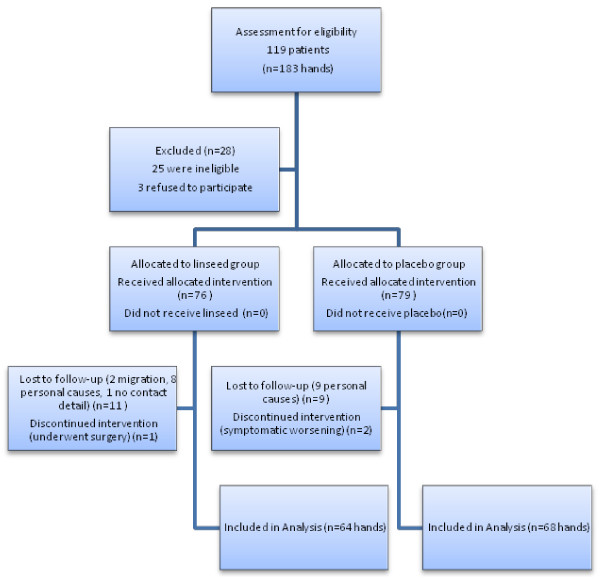
The trail flowchart.

The first enrollment was done in October 2013 and the last patient’s follow-up was completed in January 2014. A total of 119 patients (183 hands) were assessed for eligibility, and finally 100 patients (155 hands) who were eligible and gave their written informed consent were randomly assigned to drug and placebo groups (79 and 76 hands for placebo and drug, respectively). Sixty four hands in the linseed oil group and 68 hands in the placebo group completed the study. Detailed description of the patients’ enrolment, randomization and outcomes are outlined in Figure 2.

The baseline demographic and clinical characteristics of the patients are shown in Table 2. No significant differences with regard to age, gender, duration of symptoms and BMI were observed between two arms. Additionally, baseline measures for all of the outcome assessments were similar in both groups.

**Table 2:** Baseline demographic and clinical characteristics of participants in the two groups of linseed oil and placebo

**Variable**	**Placebo group (n = 79)**	**Linseed oil group (n = 76)**	***p *** **value**	**Statistical test**
Age (years), Mean(±SD)	45.01(±8.71)	42.95(±10.63)	0.227	t-test
Male/female (n)	4/64	10/54	0.069	Chi-Square
Duration(months), Mean(±SD)	13.66(±13.45)	13.56(±13.78)	0.912	Mann–Whitney
BMI (kg/m^2^), Mean(±SD)	27.03(±3.25)	25.99(±5.22)	0.173	t-test
BCTQ SYMPT (pts), Mean(±SD)	2.75(±0.62)	2.74(±0.75)	0.921	t-test
BCTQ FUNCT (pts), Mean(±SD)	2.41(±0.74)	2.61(±0.71)	0.120	t-test
Median NCV (m/sec), Mean(±SD)	36.27(±4.29)	35.54(±3.81)	0.303	t-test
Median MDL (msec), Mean(±SD)	4.16(±0.20)	4.20(±0.34)	0.384	t-test
Median CL (msec), Mean(±SD)	2.533(±0.17)	2.52(±0.14)	0.872	t-test
Median SDL (msec), Mean(±SD)	3.99(±0.26)	3.95(±0.43)	0.519	t-test

SD: standard deviation, BMI: body mass index, BCTQ: Boston Carpal Tunnel Questionnaire, SYMPT: symptom severity, FUNCT: functional status, pts: points, NCV: nerve conduction velocity, MDL: motor distal latency, CL: compound latency, SDL: sensory distal latency.

The patients were asked about their adherence to the study protocol at the follow-up visit. In the linseed oil group, only 3 patients reported some missing doses of oil and 2 patients had not used the splint as it was prescribed. In addition, in the placebo group 4 and 2 patients reported some forgotten doses of oil and inappropriate use of splint, respectively. However, all of them were analyzed in the predetermined groups. Additionally, according to the patients’ report, no rescue drug was used by them in each group.

Table 3 shows a full description of each outcome measurement changes, considering before and after trial, as well as mean differences in each group. Comparison of the mean differences between the groups showed a significant improvement in BCTQ SYMPT and FUNCT of the linseed oil group, after a 4 week period of treatment (*p* <0.001). Mean differences of these measures were 0.83 (CI 95% 0.69 – 0.99) and 0.44 (CI 95% 0.32 – 0.56), respectively.

**Table 3:** Changes in BCTQ symptoms, BCTQ function and electrophysiologic measurements, comparing mean values before and after trial within groups, and mean differences between groups

	**Study groups**	**Before (Mean ± SD)**	**After (Mean ± SD)**	***p*-value**	**Statistical test**	**Mean difference**	***p*-value**	**Statistical test**
**BCTQ SYMPT**	Linseed oil	2.74 ± 0.75	1.90 ± 0.54	<0.0001	Paired t-test	0.83 ± 0.59 ^(CI 95% 0.69–0.99)^	<0.001	Independent t-test
	Placebo	2.75 ± 0.62	2.59 ± 0.75	<0.0001	Paired t-test	0.16 ± 0.48 ^(CI95% 0.05–0.28)^		
**BCTQ FUNCT**	Linseed oil	2.61 ± 0.71	2.17 ± 0.71	<0.0001	Paired t-test	0.44 ± 0.5 ^(CI 95% 0.32–0.56)^	<0.001	Independent t-test
	Placebo	2.41 ± 0.74	2.59 ± 0.80	0.024	Paired t-test	- 0.18 ± 0.5 ^(CI 95% -0.31- -0.07)^		
**Median NCV**	Linseed oil	35.54 ± 3.81	37.92 ± 6.23	0.007	Paired t-test	2.38 ± 6.78 ^(CI 95% 0.72–4.04)^	0.034	Independent t-test
	Placebo	36.27 ± 4.29	36.22 ± 6.07	0.57	Paired t-test	0.04 ± 6.21 ^(CI 95% -1.52–1.43)^		
**Median MDL**	Linseed oil	4.20 ± 0.34	4.06 ± 0.33	<0.0001	Paired t-test	0.14 ± 0.29 ^(CI 95% 0.07–0.21)^	0.140	Independent t-test
	Placebo	4.16 ± 0.20	4.10 ± 0.35	<0.0001	Paired t-test	0.06 ± 0.32 ^(CI 95% -0.02–0.14)^		
**Median CL**	Linseed oil	2.52 ± 0.14	2.43 ± 0.23	0.004	Paired t-test	0.09 ± 0.21 ^(CI 95% 0.03–0.15)^	0.044	Independent t-test
	Placebo	2.53 ± 0.17	2.54 ± 0.32	0.145	Paired t-test	−0.008 ± 0.28 ^(CI 95% -0.08–0.06)^		
**Median SDL**	Linseed oil	3.95 ± 0.43	3.82 ± 0.34	0.032	Paired t-test	0.12 ± 0.45 ^(CI 95% 0.01–0.24)^	0.144	Independent t-test
	Placebo	3.99 ± 0.26	3.97 ± 0.36	0.053	Paired t-test	0.02 ± 0.36 ^(CI 95% -0.07–0.11)^		

SD: standard deviation, BCTQ: Boston Carpal Tunnel Questionnaire, SYMPT: symptom severity, FUNCT: functional status, NCV: nerve conduction velocity, MDL: motor distal latency, CL: compound latency, SDL: sensory distal latency, CI 95%: 95%confidence interval

In addition, regarding mean differences of both groups, a significant improvement of NCV of the median nerve was seen in the linseed oil group by a difference of 2.38 m/sec (CI 95% 0.72 – 4.04, *p* = 0.034). However, mean differences of the median nerve’s MDL and SDL in the linseed oil group showed no significant differences as compared with the placebo group (*p* = 0.14 for both items). Finally, comparison of the mean differences between the groups revealed a slight improvement in the CL of the linseed oil group (CI 95% 0.03 – 0.15, *p* = 0.044).

### Safety and tolerability

The patients in both groups were asked about positive history of dermal reactions to any topical products and if it was positive, they were instructed to test the prescribed oil on their arm for the first use.

Linseed oil was well tolerated by patients. No serious adverse effects, neither local nor systemic, were reported in the treated group. Likewise, no additional neuropathy or local neural injury was noted in electrophysiologic tests on the follow-up visit.

## Discussion

To the best of our knowledge, the present study is the first research to evaluate the effects of linseed oil on CTS. However, this herbal preparation has an ancient history of administration for different disorders, dating back to more than 10 centuries ago in ITM [22,23]. Linseed oil is a rich source of α‒linolenic acid that has been proved to possess noticeable anti-inflammatory activity [31]. In addition, further studies have confirmed its anti-inflammatory [14,15], antioxidant [16,17], and analgesic [18] properties.

Different mechanisms for its anti-inflammatory effects are explained. The linseed oil inhibits prostaglandin E_2_, leukotriene B_4_, histamine and bradykinin-induced inflammation. The oil also inhibits arachidonic acid-induced inflammation. It shows inhibition of both cyclooxygenase and lipoxygenase pathways of arachidonate metabolism [18]. Similar to other multiple herbal formulations, anesthetic properties of topical use of linseed is also shown in animal models [32]. According to the important role of inflammatory and oxidative processes in the pathophysiology of CTS [33,34], its significant effect on improving symptomatic and functional status of our patients, and slightly but statistically significant improving effect on NCV and CL can be explained partially.

Although no previous study on the efficacy of linseed oil on CTS was found, some studies evaluated other herbal preparations in the management of CTS. In a study by Zhang et al. on the efficacy of Chinese herbal therapy on CTS comparing with two other groups, 22 patients who received this therapy had a superior benefit on their visual analogue scale (VAS) score than the splint group and no significant changes of electromyography was shown [35]. Our results are in compliance with symptomatic relief of their patients with a common aspect about the presence of oleic and linoleic acid in some of these used herbs. However, this study was not blind and had a small sample size. Furthermore, they did not assess the effects of a specific herb with determined constituents and they used an uncommon drug delivery system (steaming and washing). Also, Branco et al. published an open protocol study in which CTS patients, who experienced standard treatments failure, were treated by a two-stage protocol. They were treated primarily with a specific laser acupuncture and microamps transcutaneous electrical nerve stimulation and secondarily with herbal formulas and supplements in a case-by-case manner [36]. Similar to our trial, this small size study (only 36 patients) showed a significant effect on symptomatic improvement (91.6%). However, the complexity of their management can result in practical difficulties during usage.

Another notable study, which was conducted by Eftekharsadat et al. on two groups of 30 patients, evaluated the efficacy of topical *Eremostachys laciniata* on CTS. This medicinal herb has been shown to have anti-inflammatory and antioxidant properties, like linseed oil. They showed significant improvement on palmar prehension and VAS of pain, compared with placebo. However, this plant had a local popularity, and no significant effect on electrodiagnostic criterion was reported [37].

There is also a case-series carried out by Jung et al. that assessed the usefulness of Jackyakamcho-tang on muscle spasm and pain on 81 patients complaining about these symptoms. The usefulness was reported as 72.8% for treating CTS (where only 11 CTS patients had been included). But, unlike the safety of linseed oil, they reported adverse effects in 11.1% of the total patients, 3.7% of which were severe [38].

In regard of the topical treatment for CTS, few studies were found. Of those, Jazayeri et al. published a clinical trial evaluating EMLA cream which had some beneficial effects [39]. However, this trial was not blind and EMLA cream was not as cheap and available as linseed oil.

According to ITM, patient’s temperament can affect some herbal medicine effects [40]. Therefore, as a minor assessment we evaluated the participant’s temperament. However, no relationship was found between the participant’s temperament and outcome measures. It could be explained in some ways. The used questionnaire was not designed for our target population [41] and the drug effects are possibly independent of the patient’s temperament.

Linseed oil has some important advantages as feasibility of use as a topical drug and no disturbance of ordinary daily activities (comparable with treatments such as wrist splint), conservative and noninvasive nature (regarding some invasive options such as surgery), price of the oil (1 $ for a 40 cc bottle that is adequate for a one month use), worldwide availability, and its acceptable and significant effects, especially on symptomatic and functional status and even on some electrodiagnostic parameters.

### Study limitations

Here we should highlight some limitations that we have faced with in this trial. First, insufficient number of male participants (only about 10.5% of our patients), which can affect the generalizability of our findings. In fact, according to previous epidemiologic studies, about 30% of CTS encounters were attributable to males [42]. Indeed, this is a common problem in several trials on CTS, even with male samples as small as 0-10% [43-45].

Second, as regards to reliable and valid assessment by BCTQ about subjective functional status of patients, objective functional outcome measures such as dynamometer findings could offer more reliable results.

The other important issue is to determine the transdermal penetration of the linseed oil. In this respect, the measurement of transdermal permeation of the oil and its consequent absorption into the systemic circulation can be the subject of the future studies.

And finally, the short term follow-up is another limitation. However, this is partially related to our aim as evaluation of efficacy and safety of topical linseed oil as a new complementary treatment for the management of CTS. It is also considerable that herbal medicaments might show delayed pharmacological activity. Thus, long term assessment possibly leads to better results. On the other side, unwanted effects may also be revealed in long term evaluation.

## Conclusion

It seems that linseed oil could be effective as an adjunctive therapy in the management of mild and moderate CTS, especially in improving the severity of symptoms and functional status. In addition, its effect on electerodiagnostic parameters, especially on NCV, can be considered as a valuable point.

It is, therefore, suggested that further trials of longer follow-up and larger sample size, including appropriate male/female ratio and objective functional status outcome measures are needed to confirm the value of linseed oil as a good choice for CTS and for evaluation of the involved mechanisms. Moreover, other easily applicable pharmaceutical dosage forms (such as ointment, cream, gel and patch form) can be considered in future studies.

## Abbreviations

CTS: Carpal tunnel syndrome; ITM: Iranian traditional medicine; BCTQ: Boston carpal tunnel questionnaire; NCV: Nerve conduction velocity; MDL: Motor distal latency; SDL: Sensory distal latency; CL: Compound latency; CAM: Complementary and alternative medicine; BMI: Body mass index; SUMS: Shiraz University of Medical Sciences; GC: Gas chromatography; SYMPT: Symptom severity; FUNCT: Functional status; SD: Standard deviation; VAS: Visual analogue scale.

## Competing interests

The authors declare no competing interests.

## Authors’ contributions

MHH has made substantial contributions in conception, designing, acquisition of data and drafted the manuscript. KH had contribution in designing and preformed electrodiagnostic assessments. AA had contribution in designing, preformed electrodiagnostic assessments and revised the manuscript critically for important intellectual content. AS had contribution in designing and analyzing of data. MT produced the linseed oil and performed its standardization. MH had contribution in conception and designing and revised the manuscript critically for important intellectual content. All authors read and approved the final manuscript.
